# Genetic Analysis of Choroideremia-Related Rab Escort Proteins

**DOI:** 10.3390/ijms26083636

**Published:** 2025-04-11

**Authors:** Zhuo Xing, Fuguo Wu, Eduardo Cortes-Gomez, Annie Pao, Lingqiu Gao, Avrium Douglas, Yichen Li, Joseph A. Spernyak, G. William Wong, Prashant K. Singh, Jianmin Wang, Song Liu, Yasmin Thanavala, Ian M. MacDonald, Xiuqian Mu, Y. Eugene Yu

**Affiliations:** 1 The Children’s Guild Foundation Down Syndrome Research Program, Department of Cancer Genetics and Genomics, Roswell Park Comprehensive Cancer Center, Buffalo, NY 14263, USA; zhuo.xing@roswellpark.org (Z.X.); avrium.douglas@roswellpark.org (A.D.); 2 Department of Ophthalmology, Ross Eye Institute, State University of New York at Buffalo, Buffalo, NY 14203, USA; fuguowu@buffalo.edu (F.W.);; 3 Department of Biostatistics and Bioinformatics, Roswell Park Comprehensive Cancer Center, Buffalo, NY 14263, USA; 4 Genomic Medicine Institute, Lerner Research Institute, Cleveland Clinic, Cleveland, OH 44195, USA; liy13@ccf.org; 5 Department of Cell Stress Biology, Roswell Park Comprehensive Cancer Center, Buffalo, NY 14263, USA; joseph.spernyak@roswellpark.org; 6 Department of Physiology, Johns Hopkins University School of Medicine, Baltimore, MD 21205, USA; 7 Department of Cancer Genetics and Genomics, Roswell Park Comprehensive Cancer Center, Buffalo, NY 14263, USA; 8 Department of Immunology, Roswell Park Comprehensive Cancer Center, Buffalo, NY 14263, USA; yasmin.thanavala@roswellpark.org; 9 Department of Ophthalmology, Université de Montréal, Montreal, QC H3T 1J4, Canada; 10 Genetics, Genomics and Bioinformatics Program, State University of New York at Buffalo, Buffalo, NY 14203, USA

**Keywords:** choroideremia, inherited retinal degeneration, Rab escort proteins, mouse mutants, genetic analysis, metabolism, inflammatory biomarkers

## Abstract

Choroideremia is a rare X-linked recessive retinal disorder characterized by progressive vision loss caused by retinal degeneration resulting from mutations in the *CHM* gene, which encodes Rab escort protein 1 (REP-1). In humans and mice, the Rab escort protein (REP) family consists of two members, REP-1 and REP-2, with REP-2 hypothesized to compensate for REP-1 deficiency in tissues outside the eye in choroideremia. In this study, we conducted a systematic mutational analysis of the mouse orthologs of REP-1 and REP-2. Blood analyses revealed metabolic abnormalities in the mutant mice deficient for REP-1, resembling the systemic metabolic disturbances observed in individuals with choroideremia, such as altered lipid and hemoglobin metabolism. We also observed an elevation in systemic inflammatory biomarkers in these mutant mice. Interestingly, these systemic abnormalities emerged before retinal degeneration became detectable in REP-1-deficient mice. Transcriptomic analysis of retinas isolated from REP-1 deficient mice revealed enrichment of proinflammatory signaling pathways. These results suggest important similarities between choroideremia and some forms of retinitis pigmentosa. While engineered loss of REP-2 alone caused no detectable phenotypic changes, dual deficiency in REP-1 and REP-2 resulted in lethality under both in vivo and in vitro conditions. Our findings offer novel insights into REPs and deepen our understanding of choroideremia, which may contribute to the development of new treatments for this genetic condition.

## 1. Introduction

Choroideremia, an inherited retinal degenerative disease, is characterized by progressive vision loss due to retinal degeneration [1,2]. This condition is associated with mutations in Rab escort protein 1 (REP-1). In humans, the Rab escort protein (REP) family comprises two proteins, REP-1 and REP-2, encoded by the *CHM* and *CHML* genes, respectively. REPs act as accessory proteins of Rab geranylgeranyl-transferase (RabGGTase, also known as GGTase-II), serving as chaperones for Rab proteins during their movement toward GGTaseII and thus facilitating Rab prenylation, a specific type of post-translational modifications [3].

To date, most studies on choroideremia have focused primarily on the ocular system in both human and animal models [4,5,6,7,8,9]. However, a recent report suggests that choroideremia may be more than a retinal disease, as individuals with choroideremia displayed systemic metabolic alterations [10]. In this study, we aimed to expand the analysis of the systemic impact of a REP-1 mutation by employing a mouse model with a conditional deficiency for *Chm*, the mouse ortholog of human *CHM* located on the mouse chromosome X.

Our findings revealed that, consistently with human observations, REP-1 deficiency results in metabolic alterations, establishing this important phenotype associated with choroideremia in mice. In addition, we obtained evidence that REP-1 deficiency leads to an elevation in systemic inflammatory biomarkers, the neutrophil-to-lymphocyte ratio (NLR), serum interleukin-6 (IL-6), and interleukin-17 (IL-17). We also carried out a transcriptomic analysis of retinas from choroideremia mice and identified upregulated critical proinflammatory genes and signaling pathways.

It has been hypothesized that REP-2 compensates for REP-1 deficiency in extra-ocular tissues of individuals with choroideremia [11]. To fill the key gaps in the understanding of REP, we next engineered the first mouse mutant carrying a null allele of *Chml* (the mouse ortholog of *CHML*, encoding for REP-2) and analyzed the impacts of the mutation. Furthermore, we created and analyzed a mutant deficient in both REP-1 and REP-2, assessing the impact of dual deficiency both in vivo and in vitro.

Our functional genetic analysis of the Rab escort protein family substantially expands our knowledge of the consequences of their mutations, which may have important implications for a deeper understanding of choroideremia and the facilitation of new therapeutic strategies.

## 2. Results

### 2.1. Mouse Model of Choroideremia Exhibits Metabolic Abnormalities That Mimic the Human Phenotype

Rab escort protein 1 (REP-1), encoded by *Chm* in mice, is essential for normal ocular function, and its deficiency is causally associated with choroideremia [1,6,12]. To further explore its roles mechanistically in phenotypic manifestations, we generated and analyzed mutant mice deficient for REP-1. Constitutional deficiency of REP-1 results in embryonic lethality in mice due to abnormalities in trophoblast development and vascularization [12,13]. As a result, previous research has primarily focused on heterozygous *Chm^+^*^/*−*^ females, but not *Chm*^−/−^ females nor *Chm*-/Y males. However, in humans, *CHM* mutations primarily lead to choroideremia, with the most severe clinical symptoms occurring in males [4,11]. To model REP-1 deficiency in both sexes and bypass early developmental lethality, we employed a tamoxifen-induced conditional knockout approach using floxed *Chm* mice. The mutant mice, Chm^tm1.1Seab^/H, have exon 4 of the *Chm* gene flanked by two *lox*P sites, referred to as *Chm*^flox*/*flox^ females and *Chm*^flox/Y^ males [6], collectively abbreviated as *Chm*^flox^. By crossing *Chm*^flox^ mice with B6.Cg-Tg(CAG-cre/Esr1*)5Amc/J mice [14], referred to as *CAGGCre-ER*^TM^ or *Cre* mice, we generated *CAGGCre-ER^TM^*;*Chm*^flox/flox^ females and *CAGGCre-ER*^TM^;*Chm*^flox/Y^ males, collectively referred to as *Cre*;*Chm*^flox^. Tamoxifen induction in these compound mice at the age of 2–3 months resulted in the conditional knockout (cKO) of *Chm*, referred to as *Chm*-cKO or REP-1 deficiency.

To investigate whether conditional knockout of the *Chm* gene recapitulates the retinal cell degeneration phenotype, we conducted a histological analysis of the retinas in *Chm*-cKO mice and their control (Ctrl) mice (*Chm*^flox^) at 5 months after a 3-day consecutive administration of tamoxifen. Upon confirmation of retinal degeneration in *Chm*-cKO mice (Figure S1A), resembling the retinal phenotype observed in *Chm^+^*^/*−*^ female mice [6], we set out to examine the impact of REP-1 beyond the ocular system, with a focus on systemic metabolism, because a recent study has shown significant metabolic alterations in individuals with choroideremia [10]. We measured cholesterol and hemoglobin in *Chm*-cKO and *Chm*^flox^ mice, as these biomarkers are indicative of key lipid- and hemoglobin-related pathways. Cholesterol levels were significantly lower in *Chm*-cKO mice compared with controls (Figure S1B), in line with findings of reduced cholesterol-derived metabolites in choroideremia patients. Additionally, complete blood count (CBC) analysis in *Chm*-cKO mice revealed a decline in hemoglobin levels compared with controls (Figure S1C), mirroring the decrease in related pathway metabolites observed in individuals with choroideremia [10].

**Figure 1:**
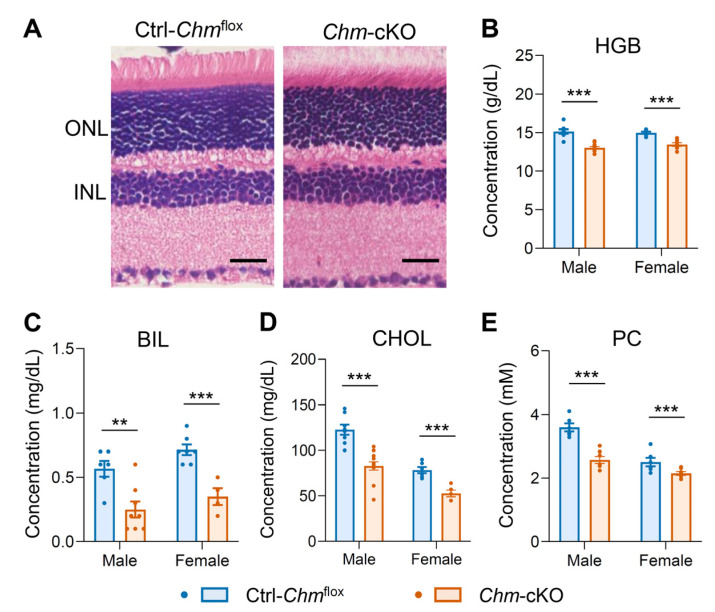
Alterations of systemic metabolic biomarkers were observed in *Chm*-cKO choroideremia mice before the detectable retinal degeneration. All analyses were conducted on mice 1.5 months after a 3-day tamoxifen administration. (**A**) A histological analysis of the retinas did not detect a change in the thickness of ONL between *Chm*-cKO and Ctrl-*Chm*^flox^ mice. ONL, outer nuclear layer; INL, inner nuclear layer. Scale bar: 25 μm. (**B**–**E**) Serum levels of hemoglobin (HGB) (**B**), bilirubin (BIL) (**C**), total cholesterol (CHOL) (**D**), and phosphatidylcholine (PC) (**E**) compared between the two groups of mice in both sexes. Sample sizes: male mice (Ctrl-*Chm*^flox^, *n* = 6–8; *Chm*-cKO, *n* = 7–12) and female mice (Ctrl-*Chm*^flox^, *n* = 6 or 7; *Chm*-cKO, *n* = 4–7). Data are presented as the mean ± SEM. Statistical analysis was performed using an unpaired two-tailed *t*-test. ** *p* < 0.01; *** *p* < 0.001.

Given that choroideremia involves progressive retinal degeneration, we sought to assess the relationship between the status of degeneration and metabolic alterations at an earlier time point. To further confirm systemic metabolic changes, we include two additional metabolites, bilirubin and phosphatidylcholine (PC), in our analysis. Histological analysis of the retinas at 1.5 months, instead of 5 months, after a 3-day tamoxifen administration revealed undetectable changes in the outer nuclear layer (ONL) thickness of the retinal photoreceptors in *Chm*-cKO mice (Figure 1A and Figure S2). However, despite the lack of detectable retinal degeneration at this early time point, statistically significant metabolic abnormalities were already evident in *Chm*-cKO mice compared with control mice, including reductions in hemoglobin, bilirubin, and cholesterol levels (Figure 1B–D). In addition, we observed a significant decline in PC (Figure 1E), a key class of phospholipids involved in lipid metabolism, which has been reported to be decreased in choroideremia patients [10] and other common inherited retinal degenerations, including retinitis pigmentosa (RP) [15]. We also compared two groups of control mice carrying *Chm*^flox^ or *Cre* and found no phenotypic difference between them (Figure S3A–D). Taken together, our results suggest that both lipid metabolism (involving PC and cholesterol) and hemoglobin metabolism (involving hemoglobin and bilirubin) may be affected by REP-1 deficiency. These results indicate that the metabolic abnormalities observed in *Chm*-cKO mice closely mirror those seen in humans with choroideremia, supporting the hypothesis that REP-1 deficiency results in systemic metabolic alterations shared between both species. Furthermore, these metabolic changes appear to occur before photoreceptor degeneration is detectable histologically.

### 2.2. Mouse Model of Choroideremia Exhibits Elevation of Systemic Inflammatory Biomarkers

**Figure 2:**
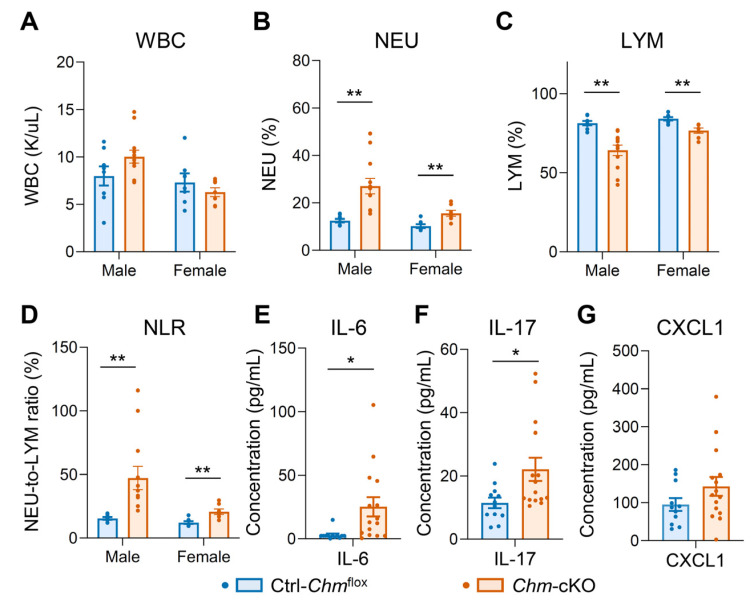
Alterations in systemic inflammatory biomarkers were observed in *Chm*-cKO mice. All analyses were conducted on mice 1.5 months after a 3-day tamoxifen administration. (**A**–**D**) Mouse complete blood count (CBC) results for white blood cell (WBC) numbers (**A**), percentage of neutrophils (NEU) (**B**), and percentage of lymphocytes (LYM) (**C**) in the WBCs, and NEU-to-LYM ratio (NLR) (**D**) between the two groups of mice. Sample sizes: male mice (Ctrl-*Chm*^flox^, *n* = 8; *Chm*-cKO, *n* = 12) and female mice (Ctrl-*Chm*^flox^, *n* = 7; *Chm*-cKO, *n* = 7). (**E**–**G**) Cytokine IL-6 (**E**), IL-17 (**F**), and chemokine CXCL1 (**G**) concentration in mouse serum. Sample size: Ctrl-*Chm*^flox^, *n* = 12; *Chm*-cKO, *n* = 15. Data are presented as the mean ± SEM. Statistical analysis was performed using an unpaired two-tailed *t*-test. * *p* < 0.05; ** *p* < 0.01. Non-significant *p*-value: WBC Male *p* = 0.1005; Female *p* = 0.3599, CXCL1 *p* = 0.1547.

CBC analysis revealed an unexpected significant elevation in the neutrophil-to-lymphocyte ratio (NLR) in *Chm*-cKO mice compared with control mice (Figure 2D and Figure S1G). NLR is an emerging biomarker linked to systemic inflammation in various diseases [16,17,18].

Because of the association between elevation of NLR and inflammation, we measured serum cytokine and chemokine levels in the mice 1.5 months after 3-day tamoxifen administration, at a time when photoreceptor degeneration had not yet been detected. Compared with *Chm*^flox^ control mice, *Chm*-cKO mice exhibited significantly elevated levels of IL-6 and IL-17 (*p* < 0.05) (Figure 2E,F), confirming conclusively that REP-1 deficiency resulted in increased systemic proinflammatory biomarkers. The alteration of CXCL1′s level was not statistically significant (*p* = 0.1547) (Figure 2G). We also confirmed that the changes in inflammatory biomarkers were not influenced by Cre activity (Figure S3E–H).

**Figure 3:**
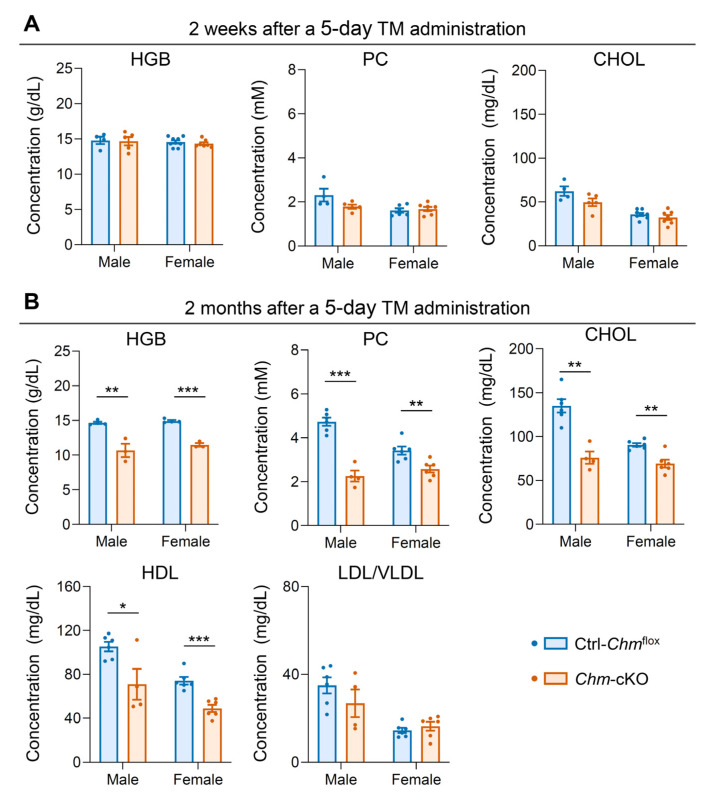
Changes in the metabolic biomarkers over time. All analyses were conducted on mice after 5 days of tamoxifen (TM) administration. (**A**) Levels of hemoglobin (HGB), phosphatidylcholine (PC), and total cholesterol (CHOL) compared between the two groups of mice 2 weeks after the tamoxifen administration. Sample sizes: male mice (Ctrl-*Chm*^flox^, *n* = 4; *Chm*-cKO, *n* = 5) and female mice (Ctrl-*Chm*^flox^, *n* = 6–9; *Chm*-cKO, *n* = 7). (**B**) Levels of HGB, PC, CHOL, high-density lipoprotein (HDL), and low-density plus very low-density lipoprotein (LDL/VLDL) compared between the two groups of mice 2 months after the tamoxifen administration. Sample sizes: male mice (Ctrl-*Chm*^flox^, *n* = 4–6; *Chm*-cKO, *n* = 3–4) and female mice (Ctrl-*Chm*^flox^, *n* = 4–6; *Chm*-cKO, *n* = 3–6). Data are presented as the mean ± SEM. Statistical analysis was performed using an unpaired two-tailed *t*-test. * *p* < 0.05; ** *p* < 0.01; *** *p* < 0.001. Non-significant *p*-value: 2 weeks HGB Male *p* = 0.8898; Female *p* = 0.4589, 2 weeks PC Male *p* = 0.1000; Female *p* = 0.6224, 2 weeks CHOL Male *p* = 0.1069; Female *p* = 0.3149, 2 months LDL/VLDL Male *p* = 0.2631; Female *p* = 0.4406.

To further understand the changes in metabolic and inflammatory biomarkers over time, we compared the mice 2 weeks or 2 months after tamoxifen administration in *Chm*-cKO and control mice (Ctrl-*Chm*^flox^). Two weeks after tamoxifen treatment, regardless of whether it was administrated for 3 days or 5 days, *Chm*-cKO mice showed significantly higher NLR compared with *Chm*^flox^ control mice (Figure S4A,B). However, at the same time point, two weeks after 5-day tamoxifen administration, no significant differences were observed between *Chm*-cKO and control mice in metabolism-related parameters, including hemoglobin, cholesterol, and PC (Figure 3A). Notably, these parameters showed a significant decline in *Chm*-cKO mice over time, two months after the treatment (Figure 3B). Moreover, we examined the levels of high-density lipoprotein (HDL) and low-density/very-low-density lipoprotein (LDL/VLDL) in the serum of mice. The results showed a significant decrease in HDL cholesterol levels in both male and female *Chm*-cKO mice compared with control mice (Figure 3B (HDL)), while there was no significant difference in LDL/VLDL levels between these two groups (Figure 3B (LDL/VLDL)). Therefore, these results suggest that a REP-1 deficiency leads to the elevation of NLR, an emerging marker for inflammatory changes that may precede metabolic alterations, both of which occur earlier than detectable retinal degeneration.

### 2.3. Retinas of Mouse Model of Choroideremia Exhibit Elevated Inflammatory Transcriptomic Biomarkers

**Figure 4:**
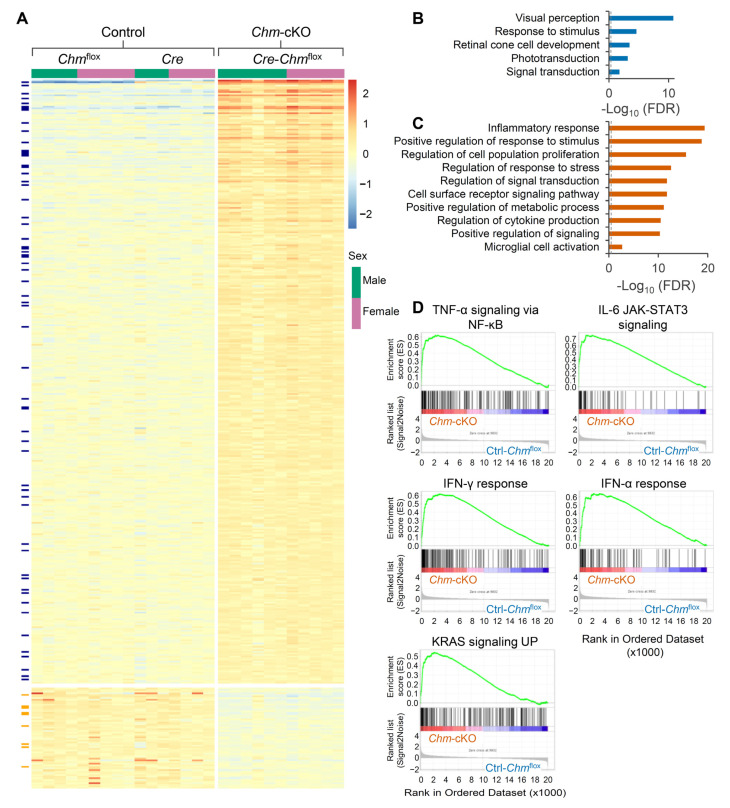
REP-1 deficiency resulted in transcriptional upregulation of inflammation related genes and transcriptional downregulation of vision-related genes in retinas. Transcriptomic analysis of the retinas from the three groups of mice 1.5 months after 3 days of tamoxifen administration. (**A**) Heatmap displays the upregulated and downregulated genes between the Ctrl-*Cre*, Ctrl-*Chm*^flox^, and *Chm*-cKO mice. Blue dashes on the left indicate genes that are upregulated in *Chm*-cKO mice and related to inflammation. Orange dashes indicate genes that are downregulated and related to visual functions. Sample sizes: male mice (Ctrl-*Chm*^flox^, *n* = 4; Ctrl-*Cre n* = 3; *Chm*-cKO, *n* = 6) and female mice (*Chm*^flox^, *n* = 5; Ctrl-*Cre n* = 4; *Chm*-cKO, *n* = 5). (**B**,**C**) Gene Ontology Biological Process (GOBP) enriched pathways of the downregulated (**B**) and upregulated (**C**) genes, identified using DAVID analysis, across the three groups of mice. (**D**) GSEA enrichment plot using the HALLMARK gene set between Ctrl-*Chm*^flox^ and *Chm*-cKO mice.

The observations of elevated NLR and serum proinflammatory cytokines in *Chm*-cKO mice prompted us to examine whether inflammation-related genes are also dysregulated in the retina of *Chm*-cKO mice at early stage of REP-1 deficiency. We performed RNA sequencing (RNA-seq) analysis on the retinas of *Chm*-cKO and two groups of control mice (*Chm*^flox^ and *Cre* mice), as previously described [19]. A total of 62 downregulated genes and 370 upregulated genes were revealed in the retinas of *Chm*-cKO mice, when compared with either *Chm*^flox^ mice or Cre mice (false discovery rate < 0.05, fold change > 1.2) (Figure 4A; Tables S1 and S2). A Gene Ontology Biological Process (GOBP) analysis revealed that the downregulated genes were enriched in pathways related to vision perception (Figure 4B). Additionally, comparison of these downregulated genes with the RetNet database (https://retnet.org/ (accessed on 20 January 2025)) identified several well-known retinal disease genes associated with REP-1 deficiency (Figure 4A,B; Table S3). Supporting our hypothesis, the GOBP analysis showed that the upregulated genes were predominantly linked to the inflammatory process (Figure 4C). Gene set enrichment analysis (GSEA) of the RNA-seq data using HALLMARK gene set revealed significant enrichment of key cytokine signaling pathways, such as IL-6/JAK/STAT3, interferon-α (IFN-α), interferon-γ (IFN-γ), and tumor necrosis factor (TNF)-α, among the most significantly upregulated genes (Figure 4D and Figure S5). An inflammation-related genes list [20] was used to identify the 59 upregulated inflammation-related genes in mice with REP-1 deficiency (Figure 4A and Figure S6; Table S4). Several of these genes have been linked to retinal conditions, including *Gfap* [21], *Csf-1* [22], and *Lif* [23,24]. Additionally, *Icam-1* and *Vcam-1* were found to be upregulated; these are known to contribute to the breakdown of blood retinal barrier, which could facilitate leukocyte adhesion and infiltration into the endothelium [25,26]. Collectively, the findings suggest that REP-1 deficiency drives increased levels of inflammatory biomarkers both intraocularly and systemically.

### 2.4. REP-2 Deficiency Leads to No Detectable Phenotypic Alterations

**Figure 5:**
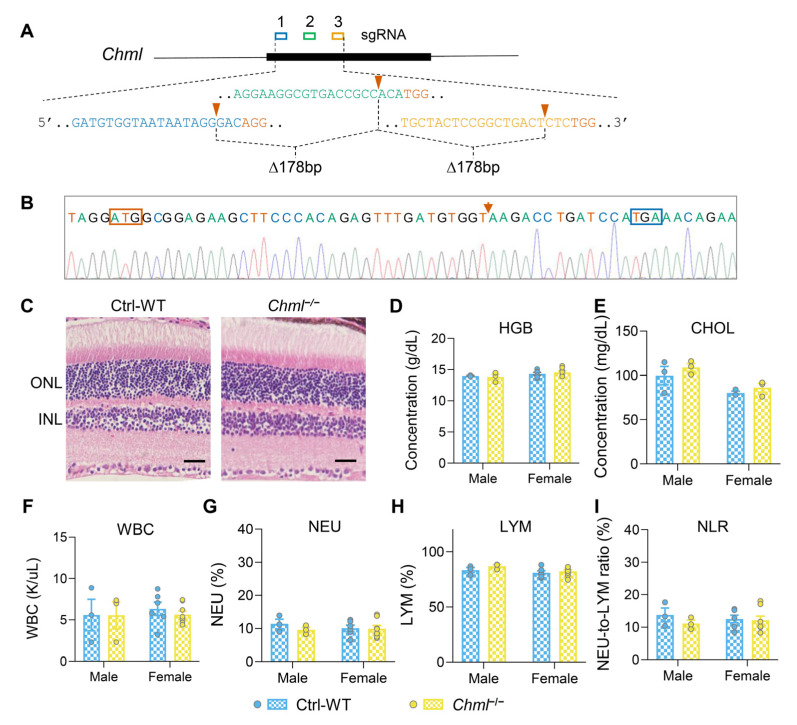
REP-2 deficient mice exhibit no detectable phenotypic alterations. (**A**) Strategy for generating *Chml* knockout mice using CRISPR/Cas9-mediated genome editing. Three sgRNA sequences are shown. Arrowheads mark the predicted cleavage sites. (**B**) Sequencing trace showing the 194 bp deletion in the *Chml* gene from Founder-1. Arrow shows the deletion break point; red rectangle indicates the start codon of the gene; blue rectangle indicates the new stop codon of the mutated allele. (**C**) Histological analysis of the retinas from the *Chml*^−/−^ mice and wild-type control (Ctrl-WT) displayed no detectable difference in the thickness of the ONL. ONL, outer nuclear layer; INL, inner nuclear layer. Scale bar: 25 μm. (**D**–**I**) Levels of hemoglobin (HGB) (**D**), total cholesterol (CHOL) (**E**), white blood cell counts (WBC) (**F**), percentage of neutrophils (NEU) in WBCs (**G**), percentage of lymphocytes (LYM) in WBCs (**H**), and NEU-to-LYM ratio (NLR) (**I**) were analyzed between the *Chml*^−/−^ mice and Ctrl-WT mice. Sample sizes: male mice (WT, *n* = 3; *Chml*^−/−^, *n* = 3) and female mice (WT, *n* = 6, *Chml^−/−^ n* = 8). Data are presented as the mean ± SEM. Statistical analysis was performed using an unpaired two-tailed *t*-test. Non-significant *p*-value: HGB male *p* = 0.7269; female *p* = 0.5226, CHOL male *p* = 0.4521; female *p* = 0.3301, WBC male *p* = 0.9890; female *p* = 0.4247, NEU male *p* = 0.3324; female *p* = 0.9000, LYM male *p* = 0.2799; female *p* = 0.5668, NLR male *p* = 0.3198; female *p* = 0.8322.

In both humans and mice, the REP family consists of two members. REP-1 has been studied extensively, particularly in regard to its role in choroideremia [6,7,27,28]. In contrast, the other member, REP-2, presumed to compensate for REP-1 deficiency outside of the ocular system [29], has remained largely unexplored. To address this gap, we used CRISPR/Cas9-mediated genome editing to create the first constitutional null allele of the *Chml* gene in mice. In this experiment, we designed three single-guide RNAs (sgRNAs) targeting the 5′ end of the *Chml* gene to generate mice with a deletion in this gene (Figure 5A). Founder mice with deleted *Chml* alleles were identified through PCR genotyping including sequencing verification. From these, we then selected seven founder mice with null alleles to generate homozygous *Chml^−/−^* knockout mice, which were confirmed by Western blot analysis (Figure S7). Among the seven founders, one specific founder mouse, Founder-1, was chosen for further analysis. This founder carried a frameshift mutation that introduced a premature termination codon, resulting in a null allele (Figure 5B). Next, we performed the cholesterol measurement and CBC tests in the homozygous *Chml^−/−^* mice and wild-type (WT) control at 7 months of age. Unlike *Chm*-cKO mice, *Chml^−/−^* mice showed no significant differences in the retina, systemic metabolism, or inflammation-related features compared with their WT controls, regardless of sex (Figure 5C–I).

### 2.5. Dual Deficiency in REP-1 and REP-2 Results in Lethality In Vivo and In Vitro

**Figure 6:**
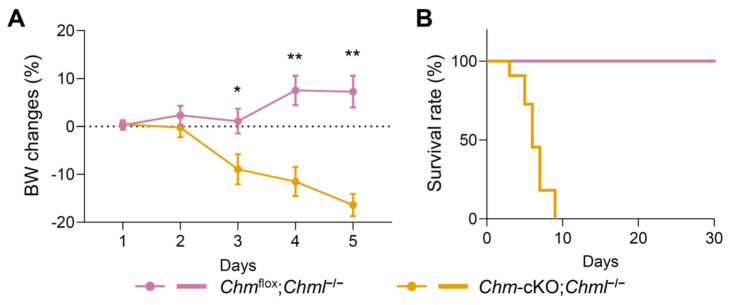
The co-deficiency in REP-1 and REP-2 led to lethality. (**A**) Body weight (BW) change in the mice following a 3-day tamoxifen administration. (**B**) Survival curve of the mice following a 3-day tamoxifen administration. *Chm*^flox^;*Chml^−/−^* mice (*n* = 7) and *Chm*-cKO;*Chml^−/−^* mice (*n* = 12). Data are presented as the mean ± SEM. Statistical analysis was performed using a multiple unpaired *t*-test. * *p* < 0.05; ** *p* < 0.01.

REP-1 deficient mice (*Chm-*cKO) exhibited significant alterations in metabolic and inflammation-related features, whereas REP-2 deficient mice (*Chml^−/−^)* showed no overt changes in metabolism or inflammation. To examine the consequence of dual deficiency in REP-1 and REP-2, *Chml^−/−^* mice were crossed with *Cre*;*Chm*^flox^ mice, resulting in the creation of *Cre*;*Chm*^flox*/*flox^;*Chml^−/−^* and *Cre*;*Chm*^flox*/*Y^;*Chml^−/−^* compound mice, collectively referred to as *Cre*;*Chm*^flox^;*Chml^−/−^*. Tamoxifen induction in these *Cre*;*Chm*^flox^;*Chml^−/−^* mice resulted in mutant mice deficient for both REP-1 and REP-2 (*Chm*-cKO;*Chml^−/−^*). These dual-deficient mice displayed significantly more severe phenotypes compared with those deficient only for REP-1. During the initial two days of tamoxifen treatment, no apparent difference was observed between the homozygous double-deficient mice (*Chm*-cKO;*Chml^−/−^*) and the control mice (*Chm*^flox^;*Chml^−/−^*). However, starting on the third day, these deficient mice exhibited a rapid decrease in body weight, accompanied by hunched postures and lethargy. By the fifth day, the mice exhibited a significant reduction in body weight, with an average loss of 16.4% (Figure 6A). This decline preceded mortality, which was observed around the seventh day (Figure 6B). Additionally, the dosage of the *Chml* gene affected the survival of the compound mutant. The *Chm* conditional knockout mice with heterozygous *Chml* knockout (*Chm*-cKO;*Chml^+/−^*) exhibited earlier mortality compared with the *Chm* single knockout mice (*Chm*-cKO), although they survived longer than the mice with homozygous *Chml* knockout (*Chm*-cKO;*Chml^−/−^*) (Figure S8).

**Figure 7:**
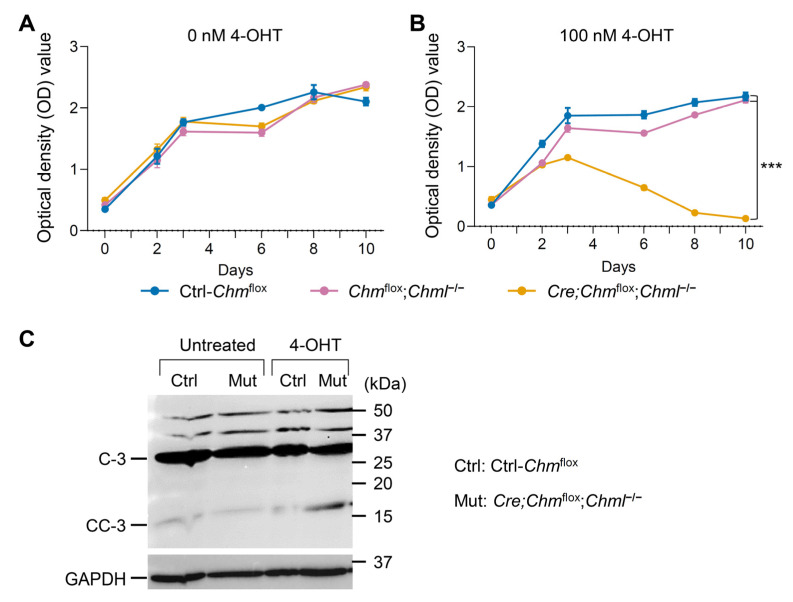
Analysis of the consequences of co-deficiency in REP-1 and REP-2 in mouse embryonic fibroblasts (MEFs). (**A**) MTT assay-based viability of MEFs with various genotypes without 4-OHT treatment. (**B**) MTT assay-based viability of MEFs with various genotypes with 100 nM 4-OHT treatment. (**C**) Western blot showing the cleaved caspase-3 (CC-3) levels of MEFs with or without 4-OHT treatment for 8 days. 4-OHT, 4-hydroxytamoxifen. C-3, caspase-3. Data are presented as the mean ± SEM. Statistical analysis was performed using a two-way ANOVA. *** *p* < 0.001.

To examine the impact of REP-1 and REP-2 deficiencies at the cellular level, mouse embryonic fibroblasts (MEFs) were isolated separately from *Chm*^flox^, *Chm*^flox^;*Chml^−/−^*, and *Cre*;*Chm*^flox^;*Chml^−/−^* embryos. The cells were treated with 100 nM 4-hydroxytamoxifen (4-OHT), an active metabolite of tamoxifen. In *Cre*;*Chm*^flox^;*Chml*^−/−^ MEFs, 4-OHT treatment induced deletion of the floxed *Chm* allele, resulting in *Chm*-cKO;*Chml* cells that are deficient in both REP-1 and REP-2. Cell viability was evaluated using MTT (3-[4,5-dimethylthiazol-2-yl]-2,5 diphenyl tetrazolium bromide) assays (Figure 7A,B; see also Figure S9). *Chm*^flox^ and *Chm*^flox^;*Chml^−/−^* cells proliferated at a similar rate, which was faster compared with that in *Chm-cKO*;*Chml^−/−^* cells, beginning on day 3 (Figure 7B). From day 6 onward, the *Chm-cKO*;*Chml^−/−^* cell counts showed significant differences compared with *Chm*^flox^ cells and *Chm*^flox^;*Chml^−/−^* cells (Figure 7B), and by day 11 after 4-OHT treatment, no viable *Chm*-cKO;*Chml^−/−^* cells remained (Figure S9). REP-1 deficiency has been linked to increased retinal apoptosis in zebrafish and mouse models [8,28,30]. In our study, elevated levels of activated caspase-3 were observed in dual-deficient MEFs by day 8, confirming that REPs deficiency also leads to apoptosis in MEF cells (Figure 7C and Figure S10). The above results indicate that the REP family is essential for survival both in vitro and in vivo in the mammalian system, extending the observations in zebrafish [8]. We confirmed that REP-2 is crucial in compensating for REP-1 deficiency, ensuring the viability of mice and the cells.

## 3. Discussion

In this study, we significantly advanced model-based investigations of choroideremia by incorporating REP-1 deficiency in both sexes, extending the analysis beyond heterozygous *Chm* knockout females and broadening the scope to address both ocular and systemic changes. We analyzed systemic metabolic alterations, proinflammatory biomarkers, and retinal transcriptome in *Chm*-cKO choroideremia mice. Additionally, we also investigated systemic changes over time following the initiation of conditional knockout of *Chm*. The results from these analyses suggest interesting similarities between choroideremia and some forms of RP [15,23,31,32].

The main efforts directed toward research on REP have historically been limited to the ocular system in subjects with choroideremia, including both humans and animal models. A major change in focus has occurred recently with the discovery of metabolic dysregulation in individuals with choroideremia by Cunha et al., whereby REP-1 deficiency now has systemic implications beyond retinal degeneration [10]. Our results showed the human systemic metabolic phenotype is recapitulated in *Chm*-cKO choroideremia mice. Specifically, we observed similar dysregulation in hemoglobin and lipid metabolism, with reductions in hemoglobin, bilirubin, cholesterol, and phosphatidylcholine in both choroideremia patients and *Chm*-cKO choroideremia mice. Notably, metabolic abnormalities were detectable 1.5 months after tamoxifen administration, even in the absence of histologically detectable photoreceptor degeneration. Thus, our results, together with the findings from the human study [10], highlight an underexplored but possible mechanistic link between choroideremia and metabolic processes. Interestingly, a recent study has also reported metabolic alterations in RP and other inherited retinal degenerative disorders, and these changes have been proposed as potential diagnostic markers [15]. These findings suggest the possibility of similar systemic metabolic alterations associated with both groups of inherited retinal degenerative conditions, choroideremia and RP.

We also found that REP-1 deficiency is associated with elevated systemic proinflammatory biomarkers. In our study, we observed an elevated NLR, a proinflammatory biomarker that has been linked to RP [33,34] and other retina-related diseases [35]. Interestingly, elevated NLR was detected as early as two weeks after tamoxifen administration, preceding both metabolic abnormalities and histologically detectable photoreceptor degeneration. The sequential emergence of these phenotypes suggests that a potential relationship exists between NLR-related inflammatory features and metabolic abnormalities. These findings also suggest early elevated NLR may be associated with the progression of retinal conditions. Furthermore, elevated levels of proinflammatory cytokines, including IL-6, were detected in the serum of *Chm*-cKO choroideremia mice. IL-6 is a key mediator in retinal disease progression, promoting vascular inflammation and impairing endothelial cell function [36,37,38]. Activation of IL-6 signaling may disrupt endothelial monolayer integrity, leading to the breakdown of the inner blood-retinal barrier [38]. The elevated level of serum IL-6 observed in *Chm*-cKO choroideremia mice suggests a potential pathogenic role, which may facilitate the recruitment and migration of immune cells to the retina and surrounding tissues [39]. The elevation of systemic proinflammatory biomarkers has also been observed in RP, although it has not been frequently reported [31,32], suggesting the possibility of similar alterations of systemic inflammation-related features between choroideremia and RP.

Our RNA-seq analysis provided the first genome-wide characterization of gene expression associated with intraocular inflammation in choroideremia, building upon previously reported evidence [5,30,40,41]. Together, these findings highlight interesting similarities between choroideremia mouse models and mouse models of *Mertk*-associated RP (*Mertk^−/−V1^* and *Mertk^−/−V2^;Tyro3^−/−V2^*) [23]. First, microglial activation was evident in both *Chm* and *Mertk* mutant mice. In *Chm*-cKO retinas, microglial and glial activation was indicated by increased expression of ***CD68*** (Tables S2 and S4) and ***Gfap*** (Table S2) genes. Additionally, an increase in IBA1-positive glial cells was observed in the retinas of six-month-old *Chm*^+/−^ female mice [30]. Second, in *Mertk*-related mutant mice, Mercau et al. identified elevated levels of inflammatory cytokines and chemokines, including macrophage colony-stimulating factor (MCSF, encoded by the ***Csf-1*** gene) and leukemia inhibitory factor (LIF) [23]. Both of the related genes were upregulated in *Chm*-cKO retinas (Tables S2 and S4). Third, the proinflammatory signaling pathways enriched in *Chm*-cKO retinas—such as IL-6/JAK/STAT3, IFN-α, IFN-γ, and TNF-α (Figure 4D)—were also among the most upregulated pathways in *Mertk* mutant retinas. The parallels in microglial activation, altered expression of inflammation-related genes, and enrichment of proinflammatory signaling pathways between *Chm*-cKO choroideremia mice and *Mertk*-related RP models [23] suggest a strong similarity in the retinal inflammation-related features at the molecular and cellular levels in choroideremia and RP. These shared characteristics likely contribute to degenerative processes in both conditions. In addition, both *Chm*-cKO choroideremia mice and *Mertk*-related RP models [23] exhibited retinal inflammation-related features that preceded detectable photoreceptor degeneration, supporting the hypothesis that inflammation may represent an early event in disease progression.

The above findings suggest that choroideremia and certain forms of RP share significant similarities in their disease characteristics. This overlap could enhance our understanding of these conditions. There is no effective treatment for many inherited retinal degenerative conditions, including choroideremia. Recently, gene therapy has emerged as a potentially effective approach and has raised hope for many inherited monogenic retinal degeneration disorders. Significant international effort has been invested and will continue to be invested to develop novel gene therapies for retinal degenerative conditions, which have eluded more traditional approaches [42,43], partly because retinas are relatively easy to access and there is prior success with RPE65 associated retinal degeneration [44]. However, despite extensive efforts, including many clinical trials, the future of gene therapy for these conditions remains uncertain [9,43,45,46]. One of the major concerns surrounding the uncertainty in the field is inflammation [9,45,46,47]. Our results showed that REP-1 deficiency drives the elevation of inflammatory biomarkers intra- and extra-ocularly, and such an elevation is sustained. These REP-1 deficiency-associated inflammation-related features may pose unusual challenges to use of gene therapy for choroideremia. The elevation of inflammatory biomarkers observed in *Chm*-cKO mice may directly or indirectly cause damage to the retina. Therefore, further mechanistic understanding of intraocular and systemic impacts of REP-1 deficiency will be critical for developing successful treatments for choroideremia and other inherited retinal degenerations, such as RP.

In this study, we generated a REP-2 deficient mouse model and investigated the impacts of REP-2 deficiency alone as well as dual deficiency of both REP-1 and REP-2 in mice. Unlike constitutional REP-1 deficiency, which leads to embryonic lethality in mice [12,13], constitutional REP-2 deficient mice can develop to term and are born healthy. Cultured *Chm*^flox^;*Chml^−/−^* MEFs exhibited normal proliferation, identical to that of the *Chm*^flox^ control. However, when REP-1 was deficient, REP-2 became essential for the survival of mouse cells. Dual-deficient REP-1/REP-2 mice succumbed approximately 7 days after tamoxifen administration. Consistent with the in vivo results, REP-1/REP-2 dual-deficient MEFs (*Chm*-cKO;*Chml^−/−^*) died about 11 days after conditional knockout of the *Chm* gene by 4-OHT treatment. These results indicate dual deficiency of REP-1 and REP-2 cause lethal phenotypes in vivo and in vitro in mice. Humans, like mice, have two REPs [8]. It is generally believed that REP-2 in non-retinal cells can compensate for REP-1 deficiency, thereby limiting the pathological effects of REP-1 deficiency to the eyes [11,29]. However, direct experimental evidence supporting this hypothesis was previously lacking until our current study. The lethality observed in REP-1/REP-2 dual-deficient mice and cells suggests that REPs are essential for survival. This is likely due to their indispensable role in facilitating the geranylgeranylation of Rab proteins, a specific form of prenylation [11]. While distinct substrates have been identified for each specific prenyltransferase [48,49], the recent discovery of a fourth type of protein prenyltransferase [50,51] and the evidence of cross-substrate prenylation [52,53] suggest the uncertainty surrounding of the prenylation specificity for particular proteins, further adding to the complexity of prenylations. Nonetheless, the lethality observed in zebrafish [8,28] and mice lacking REPs does indicate that REP-mediated Rab geranylgeranylation is an evolutionarily conserved process, and the over 400–450 million years of evolution between these species did not lead a mechanism that bypasses REP’s essentiality. In short, the essential prenylation of Rabs cannot be accomplished without REPs, underscoring their unique non-redundant role in cells.

Besides ascertaining the essential role of the REP family for survival in a mammal, our results also confirmed the functional differences in REP-1 and REP-2. In mice, the absence of REP-2 does not cause any noticeable changes when REP-1 is present. The REP-2 deficient mice grew and thrived up to the end of our experiments (7 months old) without any detectable abnormalities in retinas, metabolism, or inflammatory response. In contrast, REP-1 deficiency results in quite severe consequences, including photoreceptor degeneration, metabolic abnormalities, and elevation of inflammatory biomarkers, even in the presence of functional REP-2. The functional difference in REP-1 and REP-2 is likely due to their capacity in modulating tissue-specific Rab prenylation efficiency. Studies have demonstrated that Rab proteins exhibit varying rates of geranylgeranylation depending on whether REP-1 or REP-2 is involved [49,54,55]. Our results also expand the understanding of the relationship among REPs and survivability, advancing from the aforementioned qualitative level to a quantitative level: *Chm*-cKO;*Chml^+/−^* mice, which were deficient for the *Chm* gene but retained one copy of the functional *Chml* gene, survived longer, with a lifespan of approximately 25 days, than *Chm*-cKO;*Chml^−/−^* dual-deficient mice, which had a lifespan of approximately 7 days (Figure 6 and Figure S8). This suggests that the copy number of *Chml* impacts the lifespan of *Chm*-cKO mice quantitively.

### Future Direction

Our findings have addressed key gaps in understanding the functional roles of REPs, particularly in relation to choroideremia. This progress lays the foundation for further investigations, including the assessment of proinflammatory biomarkers in individuals with choroideremia and the evaluation of gene therapies in the *Chm*-cKO mouse model. These future efforts may not only provide deeper mechanistic insights but also pave the way for therapeutic advancements, ultimately contributing to the development of effective treatments for this rare disease.

## 4. Materials and Methods

### 4.1. Generation of Desired Mouse Mutants

The transgenic mouse strain Tg(CAG-cre/Esr1*)5Amc/J, referred to as *CAGGCre-ER^TM^*, was purchased from the Jackson Laboratory. This strain, with a C57BL/6 strain background, features a tamoxifen-inducible Cre-mediated recombination system [14]. Additionally, floxed *Chm* mice (*Chm*^tm1.1Seab^/H, referred to as *Chm*^flox^) were obtained from the European Mutant Mouse Archive/ Harwell [6]. By crossing *Chm*^flox^ mice with *CAGGCre-ER^TM^* mice, we generated the compound mutants *CAGGCre-ER^TM^*;*Chm*^flox*/*flox^ females or *CAGGCre-ER^TM^*;*Chm*^flox/Y^ males, collectively referred to as *Cre*;*Chm*^flox^.

For the induction of a null allele in the *Chm* gene, tamoxifen (Sigma, T5648, St. Louis, MO, USA) was dissolved in corn oil (Sigma, C8267) to achieve a concentration of 10 mg/mL. Tamoxifen was administered intraperitoneally at a dose of 3 mg per 40 g body weight to 2–3 month-old mice of three groups, *Cre*;*Chm*^flox^, Ctrl-*Chm*^flox^, or Ctrl-*Cre* following either a 3-day or 5-day consecutive regimen [14].

To generate a null allele of *Chml* in mice, we utilized CRISPR/Cas9-mediated genome editing. We designed three single-guide RNAs (sgRNAs) and annealed their corresponding oligonucleotides, and then, we inserted them into the *Bbs*I site of the pX330-U6-Chimeric_BB-CBh-hSpCas9 vector (Addgene plasmid #42230, Watertown, MA, USA) [56]. T7 promoters were added to the sgRNA templates via PCR, and the sgRNAs were synthesized using the MEGAshortscript™ T7 Transcription Kit (Invitrogen, AM1354, Waltham, MA, USA). Cas9 mRNA was obtained from the Washington University Core Facility. Female C57BL/6 mice underwent superovulation and timed mating, and fertilized eggs were harvested for microinjection with Cas9 mRNA and sgRNA mixtures. After overnight culturing, the embryos were transferred into pseudopregnant females. Founder mice bearing the targeted *Chml* mutations were identified through PCR genotyping and sequencing, with Western blot analysis used to confirm the elimination of REP-2 at the protein level. PCR reactions using the forward primer 5′-GGTAACATTTACCGCTATGGTG-3′ and reverse primer 5′-CTTTGGGCAAGCTTGCAGAATCCA-3′ were performed to distinguish the *Chml* mutant alleles from the WT allele.

To examine the impact of co-deficiency of REP-1 and REP-2 on survival, mice with various genotypes were administered tamoxifen from day 1 to day 3. We measured body weight starting on day 1 to monitor their response to both REP-1 and REP-2 deficiencies over time.

### 4.2. Animals

All mice were maintained in a temperature- and humidity-controlled animal facility with a 12 h light/dark cycle and had ad libitum access to a standard chow diet and sterilized water. All experimental procedures were approved by the Institutional Animal Care and Use Committee of Roswell Park Comprehensive Cancer Center. We have complied with all relevant ethical regulations for animal use.

### 4.3. Histology Analysis

Eyes from mice were fixed in 10% buffered formalin for 24–48 h and subsequently stored in 70% ethanol before paraffin embedding. Paraffin sections were stained with Hematoxylin and Eosin (H&E).

### 4.4. Analysis of Mouse Blood

Blood was collected by cardiac puncture from CO_2_-euthanized mice, which was then divided into two tubes. A 300 µL aliquot was placed in EDTA-coated tubes for CBC analysis using the ProCyte One Hematology Analyzer (IDEXX, Westbrook, ME, USA). The remaining blood was transferred to separate tubes, centrifuged to obtain serum, and analyzed for bilirubin and total cholesterol levels using the Catalyst One Chemistry Analyzer (IDEXX). Phosphatidylcholine (PC) concentration was measured using the Abcam (Cambridge, UK) PC assay kit (ab83377). High-density lipoprotein (HDL) cholesterol, low-density and very-low-density lipoproteins (LDL/VLDL) cholesterol were determined using the EnzyChrom^TM^ HDL and LDL/VLDL Assay Kit (BioAssay Systems, EHDL-100, Hayward, CA, USA). The absorbance values were read with a microplate reader (SpectraMax M2e, Molecular Devices, San Jose, CA, USA).

### 4.5. Cytokine/Chemokine Assay

Mouse serum collected as described above was aliquoted and stored at −80 °C until analysis. Cytokine/chemokine levels were determined using a customized MILLIPLEX^®^ Mouse Cytokine/Chemokine Magnetic Bead Panel (EMD Millipore, Billerica, MA, USA) on the Luminex-MAGPIX multiplex immunoassay system according to the manufacturer’s instructions, targeting CSF (colony-stimulating factor), chemokines CXCL1 and CXCL2, and cytokines IL-6, IL-10, and IL-17. Serum levels of CSF, CXCL2, and IL-10 were outside of the detectable ranges among all mouse groups tested. The data were analyzed utilizing MILLIPLEX^®^ Analyst 5.1 software (EMD Millipore, Billerica, MA, USA).

### 4.6. RNA Sequencing

After the blood collection procedure described above, the eyes were enucleated from the mice and placed in a tube with PBS on ice. The retinas were then dissected out, collected in microcentrifuge tubes containing 100 µL of RNAlater solution (Ambion, #AM7020, Austin, TX, USA), and incubated overnight at 4 °C. RNA was extracted from the retinas by using an miRNeasy mini kit (Qiagen, Hilden, Germany) in accordance with the manufacturer’s recommendations. On-column DNAse digestion was performed to remove any residual genomic DNA contamination. RNA samples were quantitated with a Qubit Broad Range RNA kit (Thermo Fisher, Waltham, MA, USA), and qualitative assessments were performed by using the 4200 Tapestation (Agilent Technologies, Santa Clara, CA, USA). RNA-seq libraries were prepared from 500 ng total RNA by using an RNA HyperPrep Kit along with RiboErase (HMR) kit (Roche Sequencing Solutions, Indianapolis, IN, USA) in accordance with the manufacturer’s instructions. Final libraries were purified by using Pure Beads and validated for appropriate size on a 4200 TapeStation D1000 Screentape (Agilent Technologies, Inc.). The DNA libraries were quantitated by using a KAPA Biosystems qPCR kit and pooled together in an equimolar fashion. The library pool was denatured and diluted to 350 pM with 1% PhiX control library added. The resulting pool was then loaded into the NovaSeq Reagent cartridge, for 100 paired end sequencing, and sequenced on a NovaSeq6000 following the manufacturer’s recommended protocol (Illumina Inc., San Diego, CA, USA). For each library, an average of 50 million paired end reads were generated.

### 4.7. Analysis of RNA-Seq Data

Paired end raw sequencing reads passed through the quality filter from Illumina Real-Time Analysis (RTA) were first pre-processed by using FASTQC (v0.11.8) (https://www.bioinformatics.babraham.ac.uk/projects/fastqc/ (accessed on 20 January 2025)) for sequencing base quality control (QC). The reads were mapped to the GRCm38 mouse reference genome and GENCODE (v25) [57] annotation database using STAR (v2.7.9a) [58]. Alignment files were indexed using samtools (v1.14) [59]. A second pass QC step was performed using alignment output with RSeQC (v4.0.0) [60] in order to examine abundances of genomic features, splicing junction saturation, and gene body coverage. Gene expression was quantified by using featureCounts (v2.0.0) [61] with the fracOverlap 0.98 option and then formatted into a raw counts data matrix. Differential expression analyses were performed by using DESeq2 (v1.36.0) [62], a variance analysis package developed to infer statistically significant differences in RNA-seq data. Genes were called differentially expressed (DE) when having a fold-change (FC) > 1.2 and false discovery rate (FDR) < 0.05 (using the Benjamini–Hochberg method to control the FDR). Downstream heatmaps were constructed using a regularized-log2 transformation (rlog function implemented by DESeq2). Subsequent pathway enrichment analyses were performed using GSEA (v4.3.2) [63] and DAVID [64]. GSEA coupled with MSigDB (v2023.2) [65] was used to examine Hallmark gene set.

### 4.8. qPCR Analysis

iScript cDNA Synthesis Kit (Bio-Rad, 1708890, Hercules, CA, USA) was used to perform reverse transcription of RNA. qPCR reactions were carried out with TaqMan Gene Expression Master Mix (Applied Biosystems, 4370048, Waltham, MA, USA) and TaqMan Gene Expression Assays (Applied Biosystems, 4453320) on the StepOnePlus system (Applied Biosystems, 43-766-00). Gene expression levels were normalized to the housekeeping gene *Gapdh*. The TaqMan assay probes used were as follows: *Gapdh* (Mm99999915_g1), *Gfap* (Mm01253033_m1), *Csf1* (Mm00432686_m1), *Lif* (Mm00434762_g1), *Icam1* (Mm00516023_m1), *Il6st* (Mm00439665_m1), *Jak3* (Mm00439962_m1), *Stat3* (Mm01219775_m1).

### 4.9. Cell Culture and MTT Assay

Mouse embryonic fibroblasts (MEFs) were isolated as previously described [66]. Embryos (13.5 days post coitum) were minced into 1–2 mm pieces and digested in 0.25% trypsin-EDTA (ethylenediaminetetraacetic acid) for 30 min at 37 °C. The cell suspension was centrifuged, resuspended, and cultured in Dulbecco’s Modified Eagle Medium (DMEM) supplemented with 10% fetal bovine serum (FBS, Gibco, Waltham, MA, USA). At ~70% confluency, cells were treated with 100 nM 4’-hydroxytamoxifen (4’-OHT) (Cayman Chemical, No. 14854, Ann Arbor, MI, USA) or left untreated for 11 days.

Cell viability was monitored via imaging and quantitatively assessed using an MTT assay with 3-(4,5-dimethyl-2-thiazolyl)-2,5-diphenyl-2H-tetrazolium bromide (Sigma, M5655). For the MTT assay, cells were seeded in 96-well plates (1500 cells/well, in triplicate) and treated with 100 nM 4’-OHT. The assays were performed on day 0, 2, 3, 6, 8, and 10. For each well, 10 μL of MTT working solution (5 mg/mL) was added and incubated for 4 h under culture conditions. Subsequently, 100 µL of dimethyl sulfoxide (DMSO) was added to solubilize the formazan crystals. Absorbance of solubilized formazan was measured at 590 nm (reference at 620 nm) using a SpectraMax M2e microplate reader (Molecular Devices).

### 4.10. Western Blots

Brain samples and MEFs were lysed in RIPA buffer containing Pierce™ Protease Inhibitor Tablets (Thermo Fisher, A32953) and phosphatase inhibitors (1 mM Na_3_VO_4_, 1 mM NaF, 1 mM phenylmethylsulfonyl fluoride, PMSF). The extracts were dissolved in Laemmli buffer and boiled at 95 °C for 5 min. Protein extracts were probed with primary antibodies against REP-1 (MyBioSource, MBS448107, San Diego, CA, USA), REP-2 (Santa Cruz Biotechnology, sc-398605, Dallas, TX, USA), CASPASE-3 (Cell Signaling, 14220, Danvers, MA, USA), or GAPDH (Santa Cruz Biotechnology, sc-32233).

### 4.11. Statistics and Reproducibility

GraphPad Prism 10 (GraphPad Software, Boston, MA, USA) was used to perform statistical analyses and generate figures. The data were analyzed using Student’s *t*-tests, multiple *t*-tests, and two-way ANOVA, depending on the specific comparisons. Results are expressed as the mean ± standard error of the mean (SEM). Details on sample sizes and significance levels are provided in the text and figure legends.

## Data Availability

RNA sequencing data reported in this paper will be deposited to the GEO database immediately upon acceptance of this manuscript. The data are available from the corresponding author on reasonable request.

## Supplementary Materials

The following supporting information can be downloaded at: https://www.mdpi.com/article/10.3390/ijms26083636/s1.
