# Supplementary material for: Genetic Analysis of Choroideremia-Related Rab Escort Proteins
Source: Int J Mol Sci. 2025 Apr 11;26(8):3636. doi: 10.3390/ijms26083636 (PMC12027379; doi:10.3390/ijms26083636)
Supplement: Supplementary file 1 [file ijms-26-03636-s001.zip › ijms-3478337-supplementary.pdf]

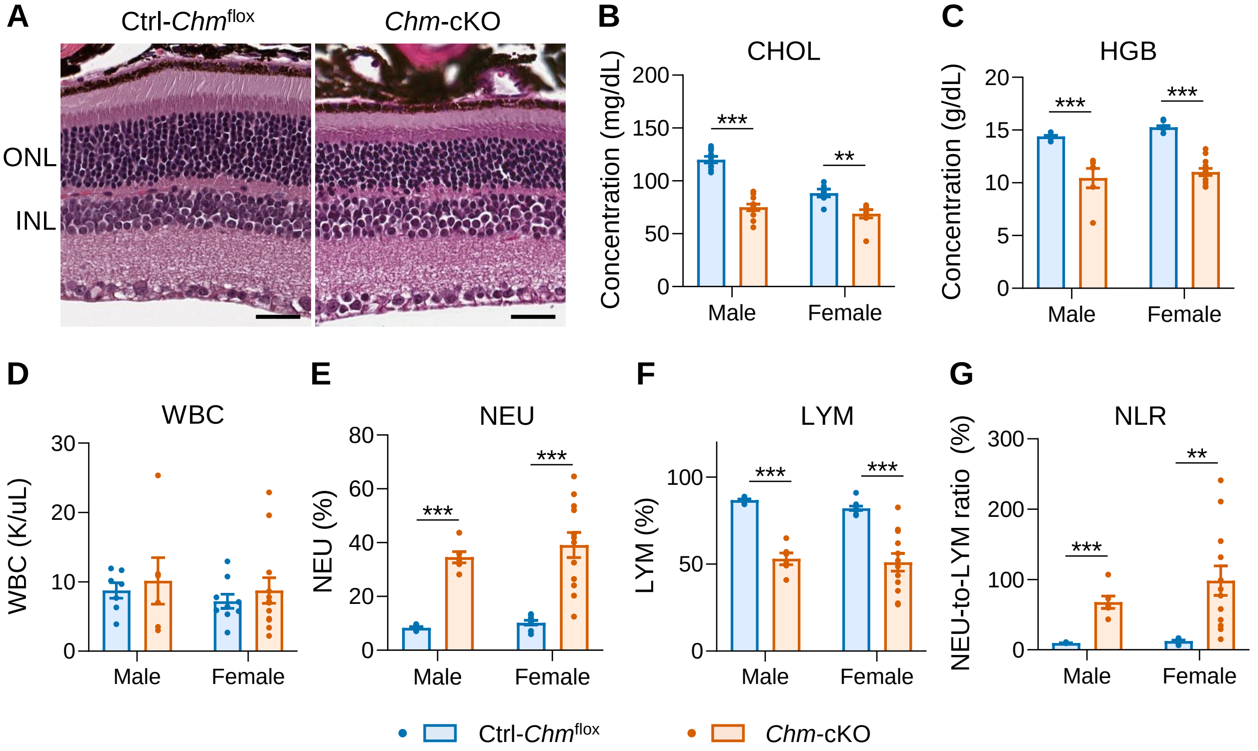

Supplementary Figure S1. Alterations in ocular layers and metabolic and inflammatory biomarkers in *Chm*-cKO choroideremia mice 5 months after a 3-day tamoxifen administration. (**A**) Histological analysis of the retinas after a 3-day tamoxifen administration showed ocular alteration in the thickness of ONL from approximately 12 nuclei in the Ctrl-*Chm*^{flox} to 6–8 nuclei in *Chm*-cKO mice at 5 months after the tamoxifen administration. ONL, outer nuclear layer; INL, inner nuclear layer. Scale bar: 25 µm. (**B**) Total cholesterol (CHOL) was measured for male mice (Ctrl-*Chm*^{flox}, *n* = 11; *Chm*-cKO, *n* = 11) and female mice (Ctrl-*Chm*^{flox}, *n* = 6; *Chm*-cKO, *n* = 8). (**C**–**G**) Complete blood count (CBC) was performed on male mice (Ctrl-*Chm*^{flox}, *n* = 7; *Chm*-cKO, *n* = 6) and female mice (Ctrl-*Chm*^{flox}, *n* = 9; *Chm*-cKO, *n* = 12). White blood cell counts (WBC) (D), percentage of neutrophils (NEU) in WBCs (E), percentage of lymphocytes (LYM) in WBCs (F), and NEU-to-LYM ratio (NLR) (G) were analyzed. Statistical analysis was performed using an unpaired two-tailed *t*-test. Data are presented as the mean ± SEM. $^{**}P < 0.01$; $^{***}P < 0.001$.

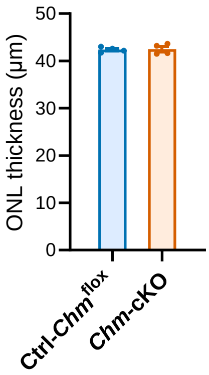

Supplementary Figure S2. Outer nuclear layer (ONL) thickness in *Chm*-cKO choroideremia mice 1.5 months after a 3-day tamoxifen treatment. Retinal ONL thickness was measured in Ctrl-*Chm*^{flox} (n = 4) and *Chm*-cKO (n = 4) mice. No significant difference was observed between the two genotypes. Statistical analysis was performed using an unpaired two-tailed *t*-test. Data are presented as the mean ± SEM.

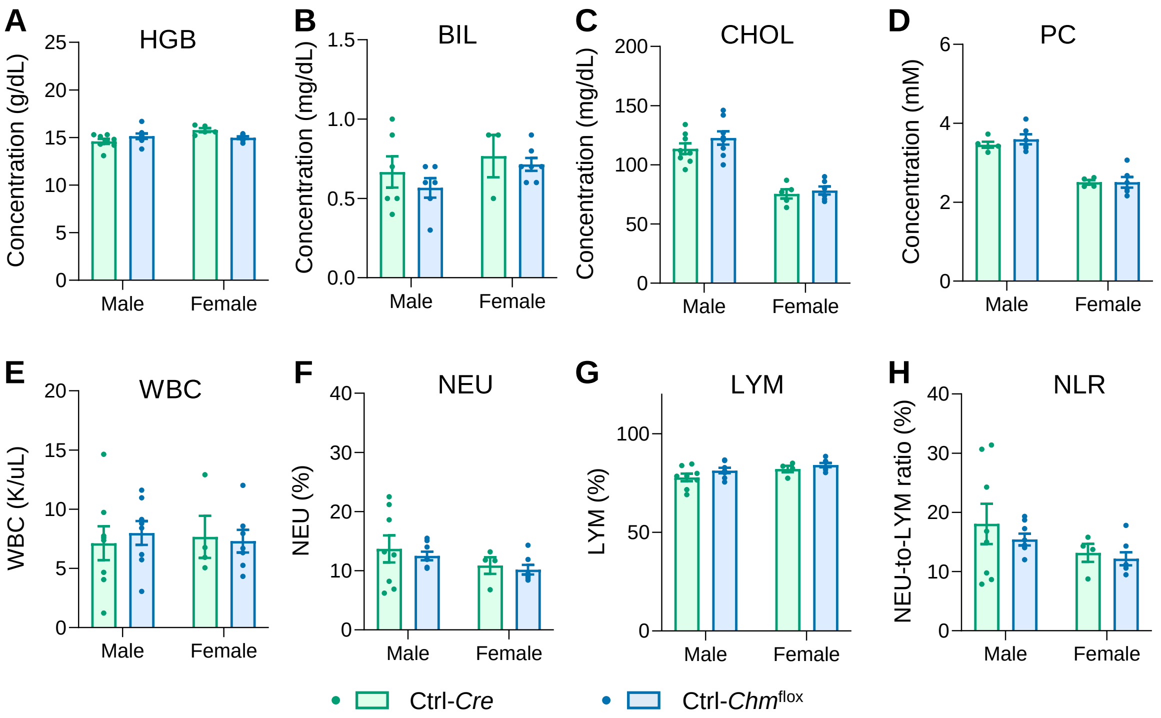

Supplementary Figure S3. No difference observed between Ctrl-*Chm*^{flox} and Ctrl-*Cre* mice. All analyses between the Ctrl-*Cre* and Ctrl-*Chm*^{flox} mice were conducted 1.5 months after a 3-day tamoxifen administration. (**A**–**D**) Serum levels of hemoglobin (HGB) (A), bilirubin (BIL) (B), total cholesterol (CHOL) (C), and phosphatidylcholine (PC) (D) were measured. Sample sizes: male mice (Ctrl-*Cre*, n = 5–8; Ctrl-*Chm*^{flox}, n = 6 or 8) and female mice (Ctrl-*Cre*, n = 3–5; Ctrl-*Chm*^{flox}, n = 6 or 7). (**E**–**H**) White blood cell counts (WBC) (E), percentage of neutrophils (NEU) (F), percentage of lymphocytes (LYM) (G), and NEU-to-LYM ratio (NLR) (H) were measured. Sample sizes: male mice (Ctrl-*Cre*, n = 8; Ctrl-*Chm*^{flox}, n = 8) and female mice (Ctrl-*Cre*, n = 4; Ctrl-*Chm*^{flox}, n = 7). Statistical analysis was performed using an unpaired two-tailed t-test.

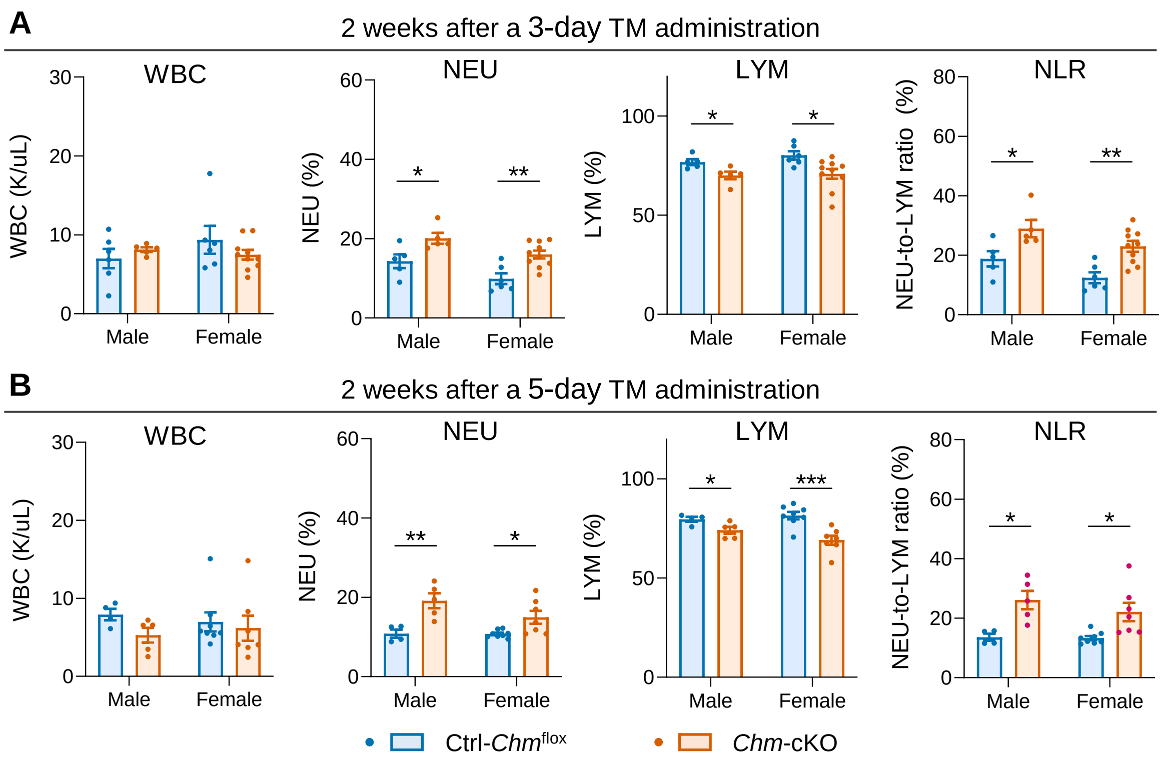

Supplementary Figure S4. Alterations in the neutrophil-to-lymphocyte ratio (NLR) in *Chm*-cKO mice 2 weeks after a 3-day or 5-day tamoxifen (TM) administration. (**A**) A complete blood count (CBC) was performed on male mice (Ctrl-*Chm*^{flox}, n = 5; *Chm*-cKO, n = 5) and female mice (Ctrl-*Chm*^{flox}, n = 6; *Chm*-cKO, n = 10) after a 3-day tamoxifen administration. (**B**) A complete blood count (CBC) was performed on male mice (Ctrl-*Chm*^{flox}, n = 4; *Chm*-cKO, n = 5) and female mice (Ctrl-*Chm*^{flox}, n = 8; *Chm*-cKO, n = 7) after a 5-day tamoxifen administration. White blood cell counts (WBC), percentage of neutrophils (NEU) in WBCs, percentage of lymphocytes (LYM) in WBCs, and NEU-to-LYM ratio (NLR) were shown. Statistical analysis was performed using an unpaired two-tailed t-test. Data are presented as the mean ± SEM. *P < 0.05; **P < 0.01; ***P < 0.001.

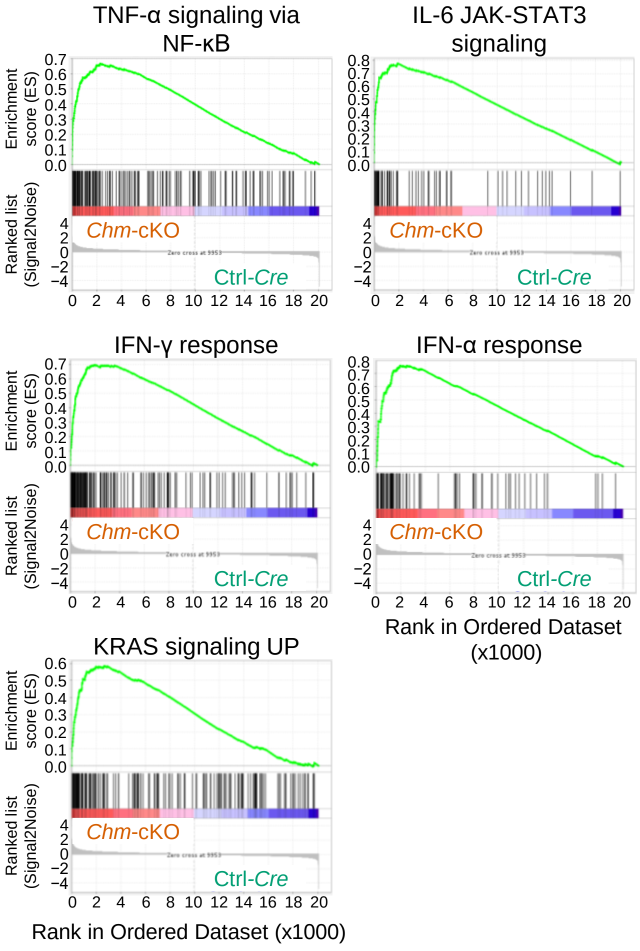

Supplementary Figure S5. GSEA enrichment plot using the HALLMARK gene set between *Chm*-cKO and Ctrl-*Cre* mice.

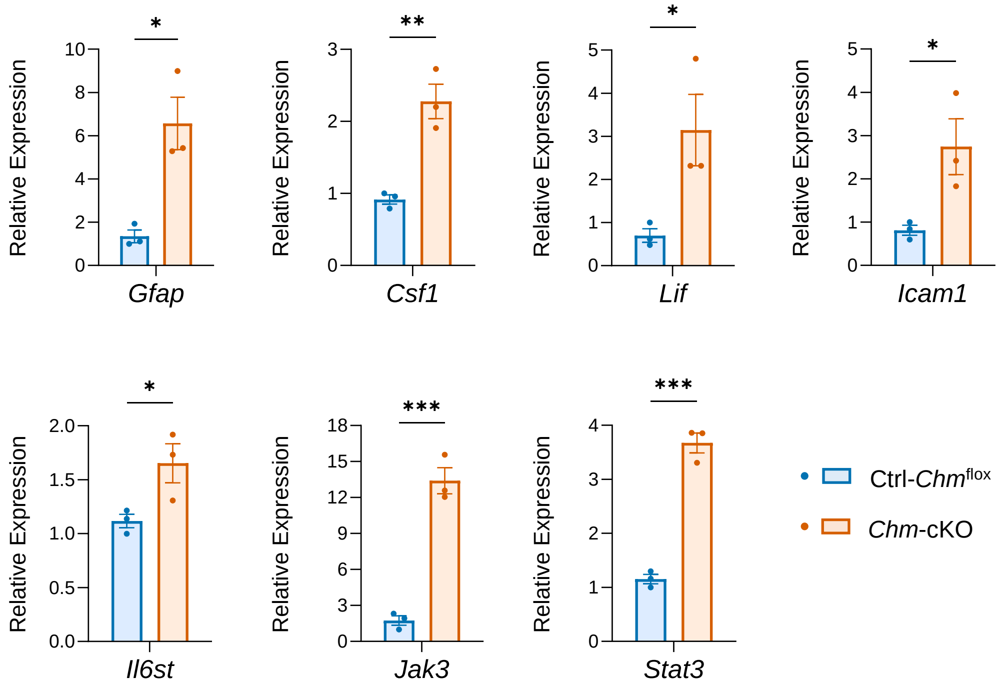

Supplementary Figure S6. Quantitative PCR validation of inflammation-related genes upregulated in *Chm*-cKO retinas relative to *Chm*^{flox} controls. Sample size: n = 3 per genotype. Statistical analysis was performed using an unpaired two-tailed t-test. Data are presented as the mean ± SEM. *P < 0.05; **P < 0.01; ***P < 0.001.

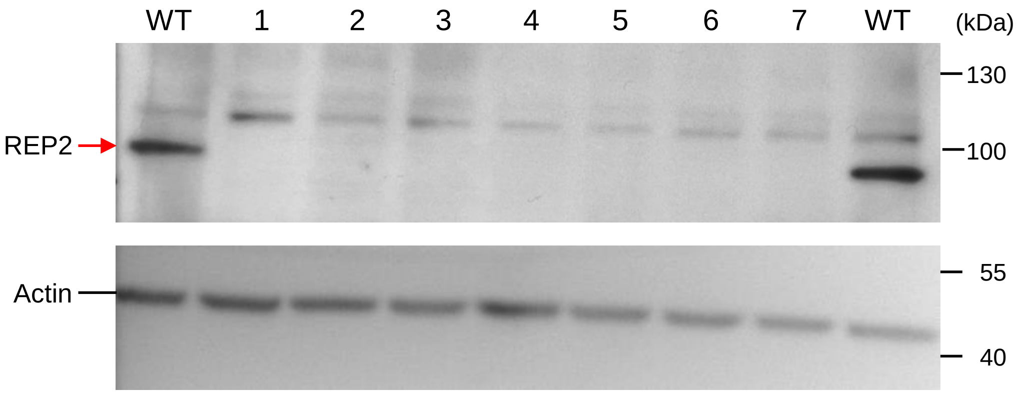

Supplementary Figure S7. Confirmation of *Chml* knockout mice. The seven homozygous *Chml* knockout mice were confirmed using Western blot analysis with an antibody against REP-2, indicated by the red arrow.

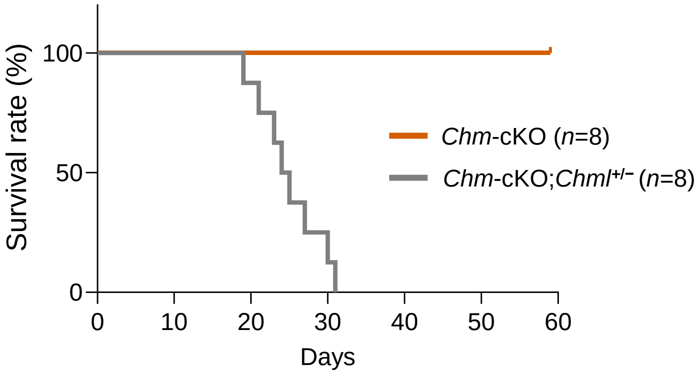

Supplementary Figure S8. Survival curve for *Chm*-cKO and *Chm*-cKO;*Chml*$^{+/-}$ mice of both sexes following a 3-day tamoxifen administration.

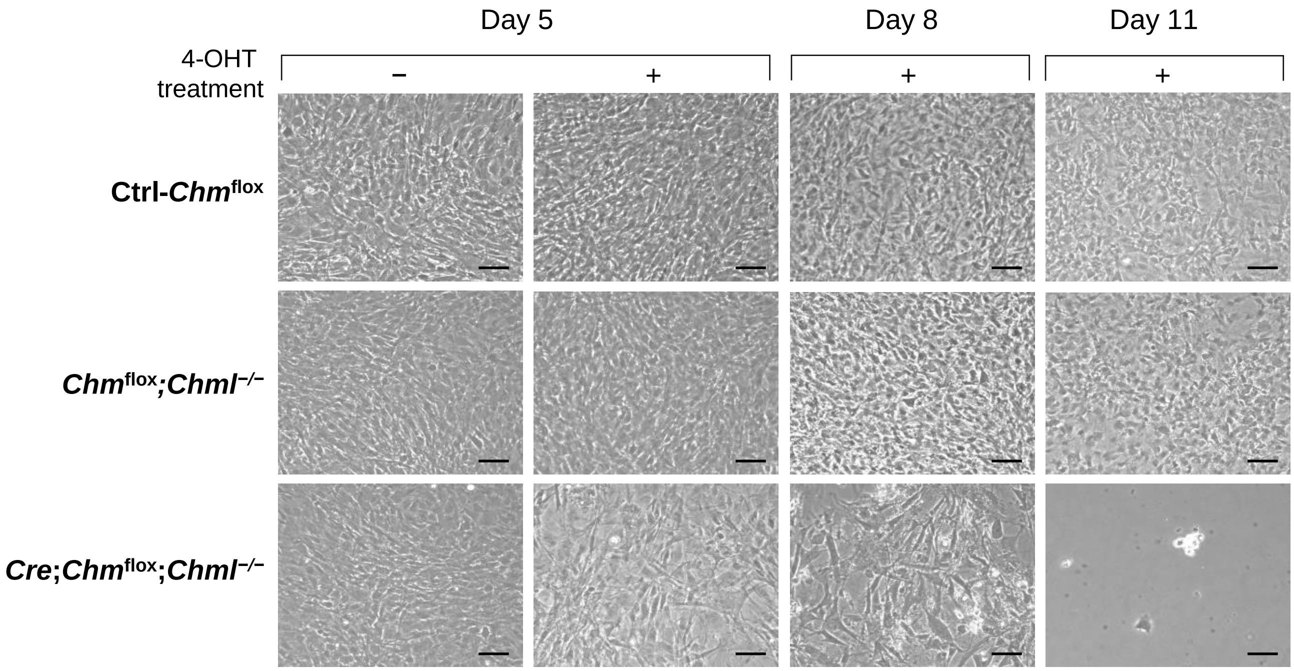

Supplementary Figure S9. Impact of co-deficiency of REP-1 and REP-2 on mouse embryonic fibroblasts (MEFs). MEFs with various genotypes were cultured and treated with 100 nM 4-hydroxytamoxifen (4-OHT) for 11 days. The images on day 5, 8, and 11 are shown. Scale bar: 100 µm.

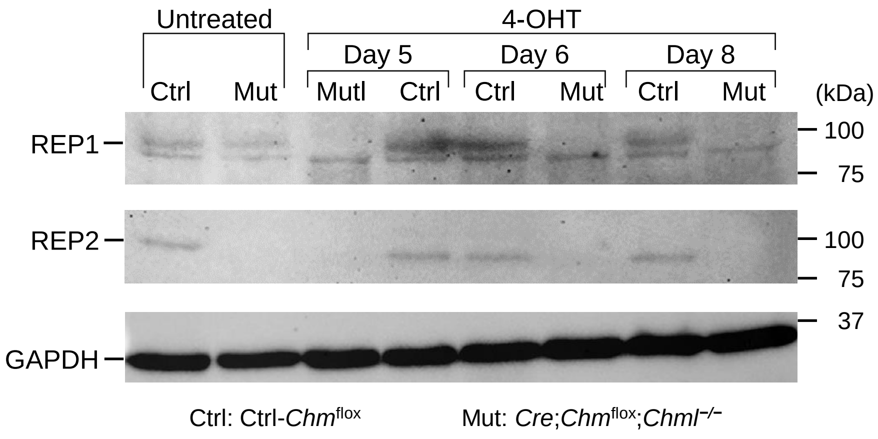

Supplementary Figure S10. Western blot analysis with the antibodies against REP-1 or REP-2 confirmed the genotypes of the MEFs employed in MTT assays and caspase-3 measurements.

Table S1. List of down-regulated genes

Actbl2	*Otop3*	*Gm15348*	*Tnfaip3*
Kif4	*Cdr1*	*Tdrd6*	*Gm22486*
Gm35715	*Mir670hg*	*Olfr1372-ps1*	*Rabgef1*
Cryaa	*Slco1c1*	*Fam3c*	*Exosc7*
Opn1sw	*Cnga3*	*Kpna2*	*Gm19840*
Arr3	*Cutal*	*H1f2*	
Gm17300	*Spc25*	*Crybb2*	
Dio2	*Gm16346*	*Pde10a*	
Gngt2	*Gzmm*	*Gm26079*	
Kcne2	*Gulo*	*Gm19466*	
Snord55	*4930554H23Rik*	*Cabp4*	
Gnat2	*Gm37376*	*Ccdc136*	
Pde6h	*Mc1r*	*Gm24265*	
Gm42697	*Ckmt1*	*Crlf3*	
Ankrd31	*Gm14091*	*Npsr1*	
Opn1mw	*Cngb3*	*Yjefn3*	
Pde6c	*Tac1*	*Tmsb10*	
Clca1	*Chm*	*Snord22*	
Vip	*Plpp2*	*Gm22489*	

Table S2. List of up-regulated genes

Edn2	*Calca*	*Hpgds*	*Gm42941*	*Msn*	*Shisa5*	*Kirrel3os*	*Shroom2*
Esr1	*C430019N01Rik*	*Adgrg6*	*Sox30*	*Gm37613*	*Vim*	*Evi2a*	*Ube2l6*
Gm20742	*6430562O15Rik*	*Tmem252*	*Ncf1*	*Hck*	*S1pr3*	*Tirap*	*Ddr2*
Cst7	*Gm10030*	*Csf3r*	*Ly86*	*Chil1*	*Cyp1b1*	*Hk1*	*Scara3*
Gm16174	*Lrrc2*	*Aqp5*	*Mt2*	*Kremen1*	*A930015P04Rik*	*Cebpa*	*4930412F12Rik*
Gm10475	*Tlr2*	*Dock2*	*Synpo*	*Asf1b*	*Kcnk6*	*Dtx3l*	*Unc93b1*
Fgf2os	*Osmr*	*Slco2b1*	*Caprin2*	*Gna14*	*Apobec3*	*Tnfrsf1b*	*Rnf213*
Lad1	*Cryzl2*	*S100a6*	*Tmem63a*	*H2-D1*	*Nckap1l*	*Ly75*	*Tcaf2*
Itgax	*Fosb*	*Tnfrsf1a*	*Lcp1*	*Gm48250*	*C230053D17Rik*	*Dcps*	*Gm12802*
Serpina3n	*Fhad1*	*C1qa*	*Tnxb*	*Trim30d*	*Bst1*	*Apobec1*	*Plpp3*
2900057B20Rik	*Ptgfr*	*Tgm2*	*Cx3cr1*	*Tmem98*	*Setdb2*	*Ildr2*	*Gm49937*
Bcl3	*Pld4*	*Icam1*	*Gm48996*	*Skap2*	*Ikzf1*	*Lrrc66*	*Vcam1*
Gm13821	*Antxr2*	*Gm4544*	*Tagln2*	*C1ra*	*Tnfaip6*	*Gm37783*	*Slc1a4*
Tnnt2	*Fam90a1b*	*Bcl6*	*Aox1*	*Itgb2*	*Selplg*	*Ikbip*	*Ano1*
Fosl1	*Btc*	*H2-Q4*	*Epha2*	*Csmd2*	*Ahrr*	*Axl*	*Sema3b*
Gm37904	*Stat3*	*Pbxip1*	*Gm15802*	*Gbp5*	*Atp1a1*	*Mindy2*	*Arhgef4*
C4b	*Cd84*	*Prag1*	*E2f6*	*Pdpn*	*Map3k1*	*Sulf1*	*Prelp*
Fgf2	*Ccr5*	*2810032G03Rik*	*Nes*	*Gm49442*	*Mlf1*	*Heg1*	*Sgcb*
Jak3	*Fam167a*	*Cfi*	*Tead3*	*Vsig10l*	*Gm22106*	*Spata6*	*R3hcc1l*
Ccdc194	*Hsd17b2*	*Cetn4*	*Zfyve28*	*Bst2*	*Atf7*	*Plekhh1*	*C4a*
A2m	*Gm42948*	*Rnf144b*	*Mcam*	*Lyz2*	*Cd68*	*Nsun4*	*Rhoj*
Dlx3	*Gm9932*	*Nupr1*	*Oasl2*	*Chl1*	*Plce1*	*Cfh*	*Akap5*
Scube1	*Nlrc5*	*Lif*	*Arap3*	*Dnmt3b*	*Clec2d*	*Ddx58*	*Impg2*
Gm43908	*Gm37717*	*Piezo1*	*Tmem132b*	*Myof*	*Atp8b1*	*Cd9*	*Gpr157*
Flnc	*Loxl4*	*Arpc1b*	*Mob3c*	*Marveld3*	*Rras*	*Gm31812*	*Tyrobp*
Steap4	*Lpin3*	*Trim34a*	*Wwtr1*	*B2m*	*Stat1*	*Zfp92*	*Creb5*
C3ar1	*Cd44*	*Hey2*	*Myo5b*	*Aldh1l1*	*Tjp3*	*Tmem150c*	*Hexb*
Cebpd	*Mpeg1*	*Gm48581*	*Phf11c*	*Irf1*	*Tmem176a*	*Kif14*	*Chst3*
Slc6a2	*Sec22c*	*Cyth4*	*Rtkn2*	*Tnip2*	*Olfm2*	*Plat*	*Ap1s2*
Eppk1	*Laptm5*	*C1qc*	*Cfap99*	*Kif28*	*Lmo1*	*Sparc*	*Plxna2*
Rfx2	*Ccno*	*Chrnb4*	*Dhh*	*Igsf11*	*Cerkl*	*Nfatc1*	*Scn7a*
Muc1	*Myo10*	*AU020206*	*Crym*	*Itgb8*	*Gpc4*	*Rnf207*	*9430037G07Rik*
Slit3	*9330188P03Rik*	*Plxnd1*	*Slc14a1*	*Arl6*	*Tubb2b*	*Pycard*	*Itgav*
Pcolce	*Gm48532*	*Ccn1*	*Mest*	*Ptprc*	*Podxl*	*Hspb6*	*Cenpe*
Gm48236	*Vwf*	*AW046200*	*Nudt6*	*Myb*	*Vwa5a*	*St3gal4*	*Ankrd6*
Gm15983	*Gm45359*	*Adamts1*	*Atf3*	*Baz1a*	*Cp*	*H2-K1*	*Gm37674*
Gm48237	*Gm47887*	*Zcchc24*	*Syt15*	*Parp3*	*Ifit1*	*Fcamr*	*Ripk1*
Clec7a	*Csf1*	*Agtpbp1*	*Gm15513*	*Itga9*	*Fzd7*	*Gm43281*	*Lfng*
Socs3	*Slc25a37*	*Ctss*	*Pls1*	*Thg1l*	*Acvr2b*	*Fas*	*Il17ra*
Gm38073	*Trf*	*C330024D21Rik*	*Ror2*	*Il6st*	*4930581F22Rik*	*Vsir*	*Gm37964*
1700061E17Rik	*Gm42759*	*Gnb3*	*Clu*	*Irf9*	*Gm44441*	*Alpk2*	*Herc6*
Gadd45b	*Cd180*	*Ahr*	*Zbtb7c*	*Gm26954*	*Gpr146*	*Gm38036*	
Tubb6	*Junb*	*Csf1r*	*Ptp4a3*	*Cnnm4*	*Tex15*	*Pmvk*	
C3	*Cela1*	*Gm45644*	*Fcrls*	*Cdh13*	*Ddx60*	*9330159M07Rik*	
Gfap	*Gadl1*	*C1qb*	*Spidr*	*Ctsc*	*Fam78b*	*Cd47*	
Fcgr2b	*Gbp2*	*Lcn2*	*Ifitm3*	*Lgals3bp*	*S100a16*	*Gbp7*	
Serping1	*Klhl29*	*Trim30a*	*Parp14*	*Helb*	*Slc41a1*	*Ipo5*	

Table S3. List of vision-related down-regulated genes correspond to the orange bars in Fig. 4A.

Down-regulated genes
Opn1sw
Gnat2
Pde6h
Opn1mw
Pde6c
Cnga3
Cngb3
Chm
Cabp4

Table S4. List of inflammation-related up-regulated genes correspond to the blue bars in Fig. 4A.

Up-regulated genes

Esr1	*C1qb*	*Cfi*
C4b	*C1qa*	*Nfatc1*
Cd44	*C1qc*	*Irf1*
Osmr	*Ctss*	*Csf3r*
Serping1	*Cd9*	*Icam1*
Jak3	*Ptgfr*	*C3ar1*
Stat3	*Cx3cr1*	*Itgb2*
Bcl3	*Stat1*	*C1ra*
Socs3	*Map3k1*	*Selplg*
Bcl6	*Il6st*	*Pycard*
H2-D1	*H2-Q4*	*Fas*
Csf1	*Csf1r*	*Il17ra*
C3	*Itgav*	*Tnfrsf1b*
Vcam1	*Ccr5*	*Atf3*
Tyrobp	*Itgax*	*Fcgr2b*
Cd47	*Tirap*	*Cd68*
Cfh	*Ptprc*	*Ripk1*
Tnfrsf1a	*Akap5*	*Cd180*
Muc1	*Tlr2*	*Cst7*
Lif	*Fosl1*	
